# Layered PEGDA hydrogel for islet of Langerhans encapsulation and improvement of vascularization

**DOI:** 10.1007/s10856-017-6004-6

**Published:** 2017-11-18

**Authors:** Giulia Marchioli, Lisa Zellner, Catarina Oliveira, Marten Engelse, Eelco de Koning, Joao Mano, Karperien, Aart van Apeldoorn, Lorenzo Moroni

**Affiliations:** 1 Department of Developmental BioEngineering, MIRA Institute for Biomedical Technology and Technical Medicine, Faculty of Science and Technology, University of Twente, Enschede, The Netherlands; 2 Department of Nephrology and Department of Endocrinology, Leiden University Medical Center, Leiden, The Netherlands; 3 Department of Complex Tissue Regeneration, MERLN Institute for Technology Inspired Regenerative Medicine, Maastricht University, Maastricht, The Netherlands; 4 Department of Chemistry, CICECO - Aveiro Institute of Materials, University of Aveiro, 3810-193 Aveiro, Portugal

## Abstract

**Abstract:**

Islets of Langerhans need to maintain their round morphology and to be fast revascularized after transplantation to preserve functional insulin secretion in response to glucose stimulation. For this purpose, a non-cell-adhesive environment is preferable for their embedding. Conversely, nutrient and oxygen supply to islets is guaranteed by capillary ingrowth within the construct and this can only be achieved in a matrix that provides adhesion cues for cells. In this study, two different approaches are explored, which are both based on a layered architecture, in order to combine these two opposite requirements. A non-adhesive islet encapsulation layer is based on polyethyleneglycole diacrylate (PEGDA). This first layer is combined with a second hydrogel based on thiolated-gelatin, thiolated-heparin and thiolated-hyaluronic acid providing cues for endothelial cell adhesion and acting as a growth factor releasing matrix. In an alternative approach, a conformal PEGDA coating is covalently applied on the surface of the islets. The coated islets are subsequently embedded in the previously mentioned hydrogel containing thiolated glycosaminoglycans. The suitability of this approach as a matrix for controlled growth factor release has been demonstrated by studying the controlled release of VEGF and bFGF for 14 days. Preliminary tube formation has been quantified on the growth factor loaded hydrogels. This approach should facilitate blood vessel ingrowth towards the embedded islets and maintain islet round morphology and functionality upon implantation.

**Graphical abstract:**

## Introduction

### Lorenzo Moroni—Reflections on career goals

I was recruited as a Ph.D. student by Isotis Orthobiologics, among the first tissue engineering companies in the world, in 2003 to develop scaffold technologies for osteochondral regeneration. The 4 years spent at Isotis were thrilling. I was embedded in a fermenting environment, where every Ph.D. student was incentivized to push own boundaries, looking for creativity and innovation while combining fundamental science with a critical view on valorization. After receiving my Ph.D. degree in 2006 at Twente University, I went to USA at Johns Hopkins University. As a post-doc in the Elisseeff lab, I focused on hydrogels and stem cells. In 2008, I was appointed the Musculoskeletal Tissue Bank R&D director of Rizzoli Orthopedic Institute, where I investigated the use of stem cells from alternative sources and the development of novel biomaterials for skeletal regeneration. These were formative years, where I could sharpen my scientific thinking, expand my hands-on skills, and move the first steps towardsc acquisition for new projects funding. From 2009 till 2014, I joined again Twente University, where I got tenured in the Tissue Regeneration department. Since 2014, I work at Maastricht University and in 2016 I became professor in biofabrication for regenerative medicine at the MERLN Institute for Technology-Inspired Regenerative Medicine.

In these years, I was infused with an interdisciplinary and intercultural mentality, where building an international network and expanding my horizons beyond the borders of my own country were pivotal to my career development. These values together with patience, perseverance, and tenacity in pursuing our scientific goals are what I try my best to transfer to my group today.
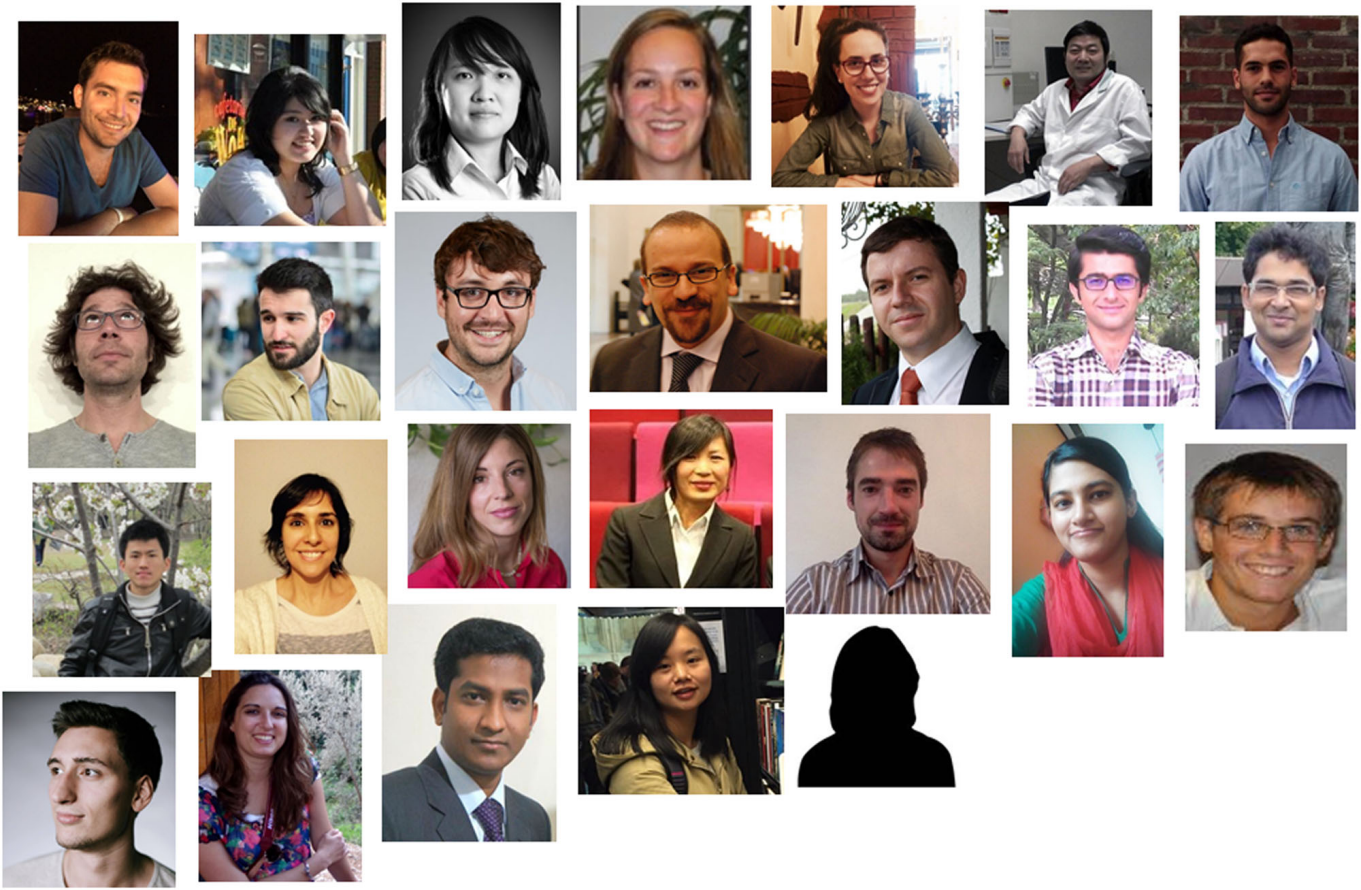


The major limitation encountered so far in tissue engineered constructs is the ingrowth of a suitable vascular bed, able to sustain in short time after transplantation the metabolic needs of the newly implanted tissue. This restriction becomes more stringent for clinically relevant implants containing sufficient amount of cells for restoring a lost function in the body, and when a highly metabolically active organ is considered [1]. Islet of Langerhans are organules in the body having one of the highest blood supply. Although islets account only for 1–2% of the total pancreatic mass, they receive up to 5–15% of the total pancreatic blood flow [2]. In clinical islet transplantation (CIT), islets are injected into the portal vein of the recipient, where they settle and start producing insulin in response to glucose stimulation [3–5]. Before injection, islets must be isolated from the pancreatic matrix by an enzymatic digestion that disrupts the extracellular matrix and the associated dense network of capillaries [6]. The loss of the islets’ vascular bed limits their ability to functionally respond to glucose stimulation and to ultimately achieve a successful transplantation [7, 8]. Due to islets loss of functionality, long term outcome for islet transplantation in the portal vein is suboptimal and less than 50% of the patients still remain insulin independent 5 years after treatment [9]. In addition to poor revascularization, other factors like instant blood mediated inflammatory reaction and complement activation (IBMIR), recurrent autoimmunity [10] and allo-immune response [11] account for islets loss of functionality in the first phases after transplantation.

Many strategies for the creation of vascular beds have been developed by several authors in the past few years. In many of these approaches, complex networks are created using 3D printing or microfluidic devices [12]. The presented results are remarkable, but these approaches are characterized by difficult applicability to clinical strategies. Some other approaches used elegant but technically complex microlithography techniques [13] which are only applicable to small scale devices.

A simpler, but equally effective approach has been attempted by stimulating blood vessel ingrowth using growth factors embedded in hydrogels [14–18]. By tuning their controlled release in time and their spatial distribution, researchers aimed at recreating the initial phases of physiological angiogenesis. It has been extensively proven that this simple but effective technique is able to induce blood vessels ingrowth in vivo [17, 18]. Such an approach has been tried with different hydrogels [15, 19–22], but the most effective ones have been demonstrated to be those introducing in their structure heparin, hyaluronic acid or binding sites for growth factor stabilization and controlled release [17, 18].

Hydrogels have shown their potential not only for growth factor delivery purposes, but have also been extensively used for islet encapsulation. Alginate has historically been the first choice for islet encapsulation, both in micro capsules [23, 24] as well for macro chamber devices for artificial pancreas [25]. The main purpose addressed by these studies was to provide a delivery vehicle for islet transplantation and containment, together with some degree of immunoprotection. The leading hypothesis behind these studies was that a properly tuned hydrogel mesh around the islets could prevent antibodies and cytokines to interact with the embedded tissue, and consequently, the recurrent attack of the immune system to allogeneic transplanted islets [26]. Such an approach has the overall aim to dismiss conventional immunosuppression regimen by substituting it with a shielding capsule which could prevent antibodies and cytokines to interact with the embedded tissue [24]. Finding a balance between proper insulin and nutrient diffusion to the islets and impeding antibodies and cytokines to permeate through the protection capsule has proven to be a very challenging task. PEGDA hydrogels have the advantage of a very defined and tunable molecular weight and chemical functionalization. Immunoprotection strategies using PEGDA functionalization have been attempted by creating a conformal coating of PEGDA on the islet surface [27–29]. Reactive groups like N-hydroxy-succinimide (NHS) or succinimidyl-valeric-acid (SVA) have the potential to react with amino groups exposed on proteins on islet’s surface and create a conformal, single molecule thick coating, on the islet itself [30].

Literature has demonstrated the immunoprotective properties of conformal islet coating by PEGylation, without the need for embedding them in micrometer sized hydrogel capsules [28]. This approach could minimize limitations arising from nutrient diffusion impairments to the islets while maintaining the immunoprotection concept.

This study presents a combination of all the aforementioned concepts, merged into a single multifunctional construct. A combination of PEGDA hydrogels for islets embedding acts as a vehicle for islets delivery and retention, while a PEGDA based layer enriched with thiolated-gelatin and extracellular matrix proteoglycans offers a cell adhesive environment for enhanced attraction of blood vessels by the controlled release of pro-angiogenic growth factors. In a similar approach, the PEGDA layer for islet embedding is reduced to a conformal coating of PEGDA covalently bound to the islet surface proteins. This second approach attempts to reduce nutrient diffusion limitations by using a thinner PEGDA layer and could at the same time provide immune-protective properties to the embedded tissue. These coated islets are then embedded in the previously mentioned hydrogel containing thiolated glycosaminoglycans for the induction of vascularization.

**Fig. 1:**
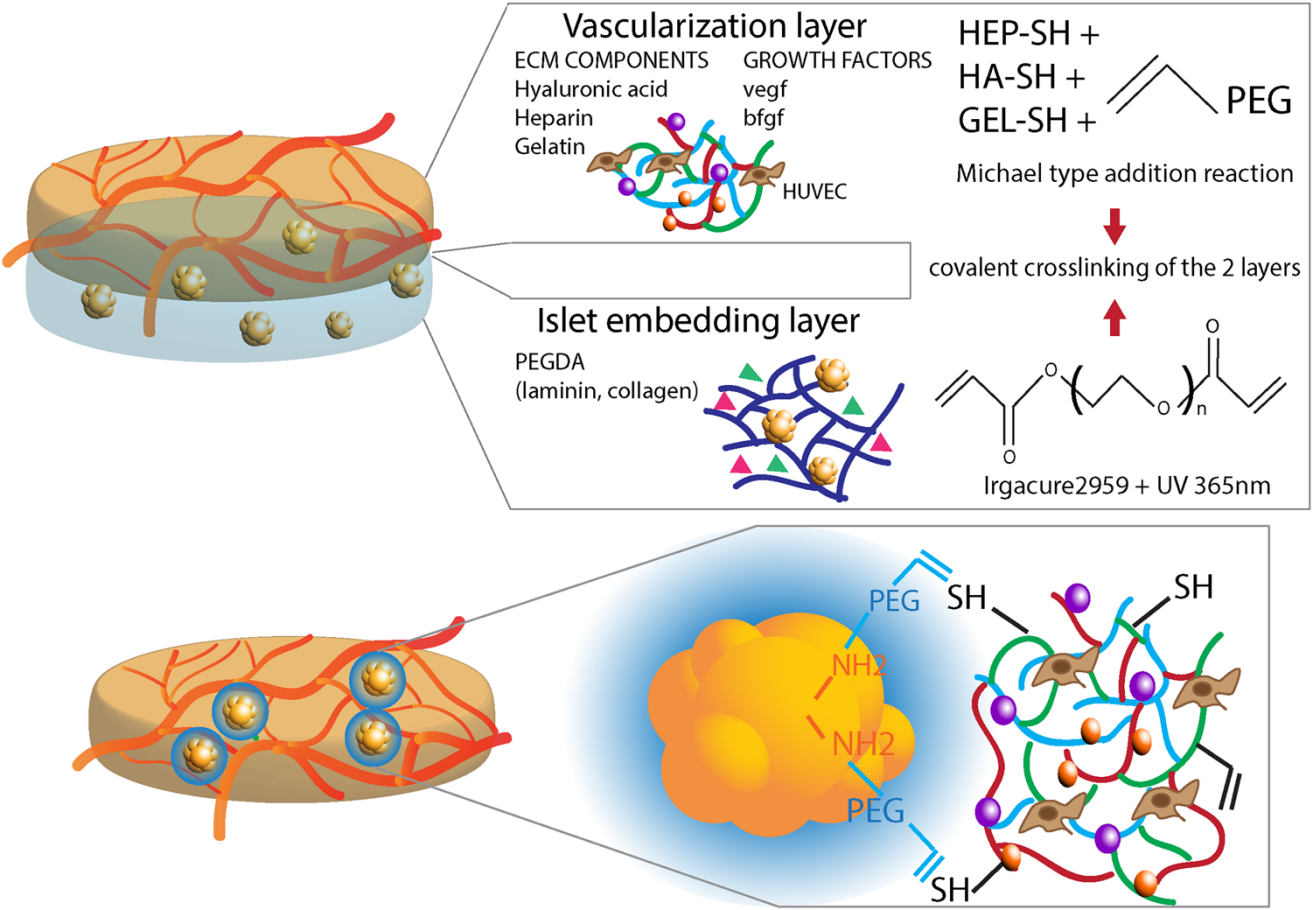
Concept of the bilayered constructs **a** Vascularization hydrogel based on Michael type reaction of acrylate groups on PEG with thiol-modified heparin, thiol-modified gelatin and thiol-modified hyaluronic acid. These ECM molecules are able to provide an adhesion cues for endothelial cells and act as a controlled release compartment for growth factor delivery. This layer can be covalently crosslinked with a PEGDA based embedding layer for islet of Langerhans encapsulation. Collagen IV or laminin can be mixed within the hydrogel mesh to create a more favorable environment for islet of Langerhans. **b** Conformal coating model where free amino groups exposed on proteins on the surface of islets are covalently modified by PEG chains. The free acrylate group on the other extremity can react with the thiol groups present in the vascularization hydrogel via Michael type reaction

A distinctive feature of such a construct is the fact that, in both cases, the two layers are covalently bound to each other by mean of free acryl groups on the surface of both hydrogels (first approach, Fig. 1a) or islets surface (second approach, Fig. 1b), creating in this way a unique, multilayered, and multifunctional construct for revascularization of transplanted islets.

## Materials and methods

### Islet of langerhans and MIN6 cell culture and pseudo-islets formation

Isolated human islets were kindly provided by the LUMC Leiden (after informed donor consent and only if the material could not be used for clinical transplantation). Islets were cultured in CMRL medium (Cellgro) supplemented with 10% FBS and 1% Pen/Strep [31], 10 mM Hepes (Gibco), 10 mM Glutamax (Gibco) and 10 mM Nicotinamide (Sigma-Aldrich).

MIN6-B1 cells, derived from a mouse insulinoma, were kindly provided by Dr. P. Halban, University Medical Center, Geneva, Switzerland. Cells were cultured in Dulbecco’s Modified Eagle’s Medium (DMEM, Gibco) with 10% (v/v) FBS (Lonza), and antibiotics (100 U/mL penicillin, 100 mg/mL streptomycin, Gibco). Beta-mercaptoethanol (Gibco) was freshly added at every medium change. Cells were cultured in standard culture conditions at 37 degrees, 5% CO_2_ in humidified atmosphere.

MIN6-B1 cells were used for the preparation of cell aggregates as previously reported by our group [32]. Briefly, cells were cultured in sterile agarose microwells (200 µm diameter) fabricated by pouring sterile agarose (3% w/v UltraPureTM Gibco Invitrogen) on top of a pre-sterilized, pre-fabricated polydimethylsiloxane (PDMS) mold with micropillars of 200 µm in diameter. One million MIN6 cells were seeded on each agarose chip and cultured for two days to allow for aggregate formation. So formed pseudo-islet could be easily removed from the chip by flushing it with some medium and used for further experiments.

### Islet of Langerhans/MIN6 pseudo-islets embedding in polyethylene glycol diacrylate gels

MIN6 pseudo-islets were resuspended in a sufficient amount of 5% w/v poly(ethylene glycol diacrylate) (PEGDA) with a molecular weight of 5000 (Laysan) in PBS (Gibco). A 10% w/v solution of Irgacure 2959 (Basf) in 70% Ethanol was prepared and 10 µl added to 1 ml of PEGDA solution prior to crosslink. Each sample was prepared using 50 µl of the precursor PEGDA, additioned with the photocrosslinker, and contained on average about 1400 MIN6 pseudo-islets.

For samples containing human collagen IV (Merck Millipore) or human laminin (Sigma-aldrich), a 20 µg/ml stock solution of protein was prepared in sterile PBS (Gibco) and diluted to the working concentration of 10 µg/ml by using 10% w/v concentrated PEGDA solution prior the addition of Irgacure (Basf). MIN6 pseudo-islets were resuspended in the solution, 50 µl samples were pipetted in a 96 well plate, and exposed to 356 nm UV light for 10 min at 5 cm distance from the light source with an intensity of 8.2 mW/cm^2^.

In case a bi-layered construct was prepared, the 50 µl PEGDA sample containing aggregates was crosslinked on top of a pre-gelled 50 µl vascularization gel.

### Glucose induced insulin secretion test

Insulin secretion after glucose stimulation was tested on either islets of Langerhans or MIN6 pseudo-islets by incubating them in Krebs buffer (115 mM NaCl, 5 mM KCl, 24 mM NaHCO_3_ Sigma) supplemented with 2.2 mM CaCl_2_, 20 mM HEPES (Gibco), 2 mg/mL bovine serum albumin, and 1 mM MgCl_2_ at pH 7.4 In case of testing MIN6 pseudo-islets functionality, theophylline 10 mM (Sigma) was added. This buffer was split into low (1.67 mM) and high glucose (16.7 mM) buffer. Cells were washed and pre-incubated for 90 min in low glucose buffer. Subsequently, cells underwent a series of 45 min incubation in 250 µl of either low glucose, high glucose, and finally again low glucose buffer, alternated by washings. Two hundred and thirty microlitres of glucose-induced insulin secretion test samples were taken after each incubation and the amount of insulin secreted by the cells upon glucose stimulation was quantified by using either a human (for islet) or mouse (MIN6 pseudo-islets) insulin ELISA kit (Mercodia).

### Islet of Langerhans PEGylation and imaging of the conformal PEGDA coating

Islets (1250 islet equivalent per sample) were washed 3 times in PBS and incubated for 1 h in a 12.5% w/v solution of NHS-PEG-Biotin (Laysan) in PBS (Gibco) at pH 8, additioned with 5 mM glucose. Islets were washed, fixed with 10% w/v formalin solution for 3 min, and subsequently incubated for an additional hour with a 20 µg/ml solution of Streptavidin-FITC (Sigma-aldrich) protecting from light [30]. PEGylated and control islets were imaged using an EVOS digital microscope (EMS) equipped with an EVOS light cube (EMS) for GFP imaging.

### Islet of Langerhans PEGylation and embedding in the vascularization hydrogel

Five hundred islet equivalent for each sample were washed with PBS supplemented with 5 mM glucose and incubated for 1 h in 350 µl of a 5% w/v solution of PEG-SVA-Acryl (laysan) in PBS additioned with 5 mM glucose [30]. Subsequently, islets were washed and sorted into 96 wells. Free floating PEGylated islets were further cultured in CMRL complete medium. PEGylated islets were also embedded within the vascularization gel by resuspending them in the gelin/heprasil/extralink mixture according to the specification by the manufacturer and allow for spontaneous crosslinking at room temperature prior to medium addition.

### Vascularization hydrogel

A commercially available product, HyStem®-HP Hydrogel Kit (ESI BIO), was used as a base for the vascularization gel. The kit provides thiol-modified gelatin (Gelin), and a combination of thiol-modified heparin and thiol-modified hyaluronic acid (Heprasil) to be crosslinked upon addition of polyethylene glycol diacrylate (Extralink). The hydrogel can be used as a matrix for growth factors delivery and cell attachment [17, 18].

Kit components were reconstituted according to the manufacturer’s instruction. Only the Extralink vial was reconstituted at a double concentration than the recommended one. All components were mixed according to Table 1. Typically, 400 µl of hydrogel precursor were prepared for each condition and 75 µl samples were used in each experiment. Basic fibroblast growth factor (bFGF) and vascular endothelial growth factor (VEGF) (Peprotech) were diluted in PBS (Gibco) with addition of 1% Bovine Serum Albumin (BSA, Sigma) to a working solution of 10 µg/ml and used for further dilution upon hydrogel preparation. Hydrogel gelation was induced by the Extralink crosslinker, and gels were left for approximately 1 h to allow for complete gelation.

**Table 1:** hydrogel preparation at different growth factor concentration according to the manufacturer’s instructions. bFGF and VEGF working solution are both 10 µg/ml

	Extralink (µl)	Gelin (µl)	Heprasil (µl)	PBS (µl)	bFGF (µl)	VEGF (µl)
200 ng/ml VEGF	40	160	160	32	–	8
500 ng/ml VEGF	40	160	160	20	–	20
100 ng/mlbFGF	40	160	160	36	4	–
200 ng/ml VEGF + 100 ng/ml bFGF	40	160	160	28	4	8
500 ng/ml VEGF + 100 ng/ml bFGF	40	160	160	26	4	20

### Human umbilical vein endothelial cells culture (HUVEC) and tube formation assay

HUVECs (Lonza) were used as a model for testing in vitro activity and vascularization potential of growth factor loaded hydrogels. HUVECs were cultured in EBM-2 basal medium (Lonza) supplemented with the provided growth factor bullet kit (EGM-2, Lonza). Cells from passage 2 to passage 5 were used for tube formation assay. Cells were stained with DiI (Invitrogen) according to the manufacturer’s specifications and incubated for 20 min at 37 °C. After incubation, cells were washed with medium containing 2% serum. Cells were seeded on top of a preformed hydrogel at a density of 15’000 cells/construct in a 96 well plate [33]. Growth factor concentration in the hydrogel were: 100 ng/ml basic fibroblast growth factor (bFGF, Peprotech), 200 and 500 ng/ml vascular endothelial growth factor (VEGF, Peprotech), combination of 100 ng/ml bFGF with either 200 or 500 ng/ml VEGF. Control conditions were 75 µl hydrogel without additional growth factors and 75 µl growth factor reduced Matrigel (BD). EGM-2 medium used for tube formation assay was prepared without the addition of VEGF or bFGF, so that the only source of growth factor was the hydrogel only. Constructs were imaged after 24 h using a BD pathway (BD) at 4× magnification to measure the extent of the tube network formed on the surface of the gel using ImageJ software (NIH). The ImageJ software defines and quantifies parameters of the tubular network. Master segments are defined as the elements of the network delimited by two junctions or branching points in the network and calculates their length in pixels. Nodes are defined as the branching points connecting two segments, of which one is not involved in the formation of another junction (dead end). Junctions refer to branching points connecting two or more master segments. Moreover, two more elements that describe the formed tubular structure are the mean mesh size (in pixels) of the area delimited by master segments; and the number of isolated segments, meaning the number of isolated segments on the picture, which are not incorporated within the network.

### Growth factor release study

Hydrogels with different growth factor concentrations (Table 1) have been prepared as previously described. 500 µl of PBS (Gibco) with 0.1% Bovine Serum Albumin (BSA, Sigma) were added to each sample and incubated at 37 C. Samples have been taken and the PBS/BSA 0.1% refreshed upon sample withdrawal. Growth factor release was measured by using a bFGF or VEGF ELISA kit (R&D) according to the manufacturer’s protocol.

### Statistical analysis

Statistical significance between groups was analyzed by performing a One-way ANOVA followed by a Bonferroni post-hoc test using IBM SPSS statistic 23 software. Data is expressed as mean ± standard deviation and significant differences are indicated with * (*p* ≤ 0.05).

## Results

Bi-layered structures could be easily fabricated by crosslinking PEGDA gels on top of a preformed vascularization gel. The presence of free thiol groups on the surface of the first hydrogel layer allowed for a covalent crosslinking of the islet encapsulation layer on top of the vascularization layer. Such bi-layered hydrogels were mechanically stable and could be easily handled as a single construct. Similar results were achieved in the second approach where islets were modified with a conformal coating of bifunctional PEG, where the SVA group reacted with free amino groups exposed on the surface of the islets and the acryl group on the other extremity of the PEG chain was available for the reaction with acryl groups in the hydrogel matrix. Both approaches, schematically represented in Fig. 1, combine the favorable properties of non-adhesive and cell adhesive layers, and can be independently tuned on the specific cell type that will be embedded within the gel. In both cases it was possible to combine the dual layer approach in a single mechanically stable construct, in which the two layers were able to crosslink with each other by establishing covalent binding.

**Fig. 2:**
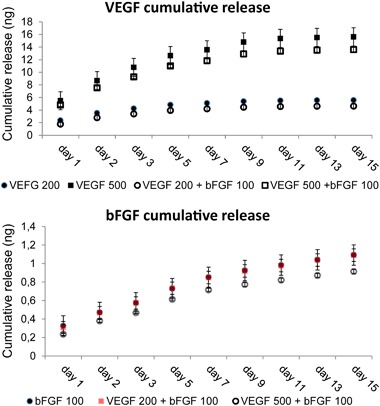
Release profile of VEGF and bFGF (different concentrations and combinations) from the PEGDA/thiolated-Hyaluronic acid/gelatin/heparin hydrogels for a release period of 14 days

The vascularization layer allowed for embedding and sustaining the release of growth factors for a period of 14 days (Fig. 2). No burst release was shown in the first day of incubation both for bFGF and VEGF. The combined release of two growth factors from the same hydrogel did not show any difference in the release kinetic compared to the same growth factor being released alone. In a period of 14 days, only about 1 ng was released from the 100 ng/ml bFGF loaded hydrogels, accounting for about a seventh of the total growth factor loading. In case of VEGF loaded hydrogels, about 4 ng were released from the 200 ng/ml VEGF or 200 ng/ml VEGF + 100 ng/ml bFGF, accounting for a fourth of the loaded amount. In case of the 500 ng/ml VEGF and 500 ng/ml VEGF + 100 ng/ml bFGF, a third (~13 ng) of the originally loaded amount was cumulatively released in a 14 days investigation time.

**Fig. 3:**
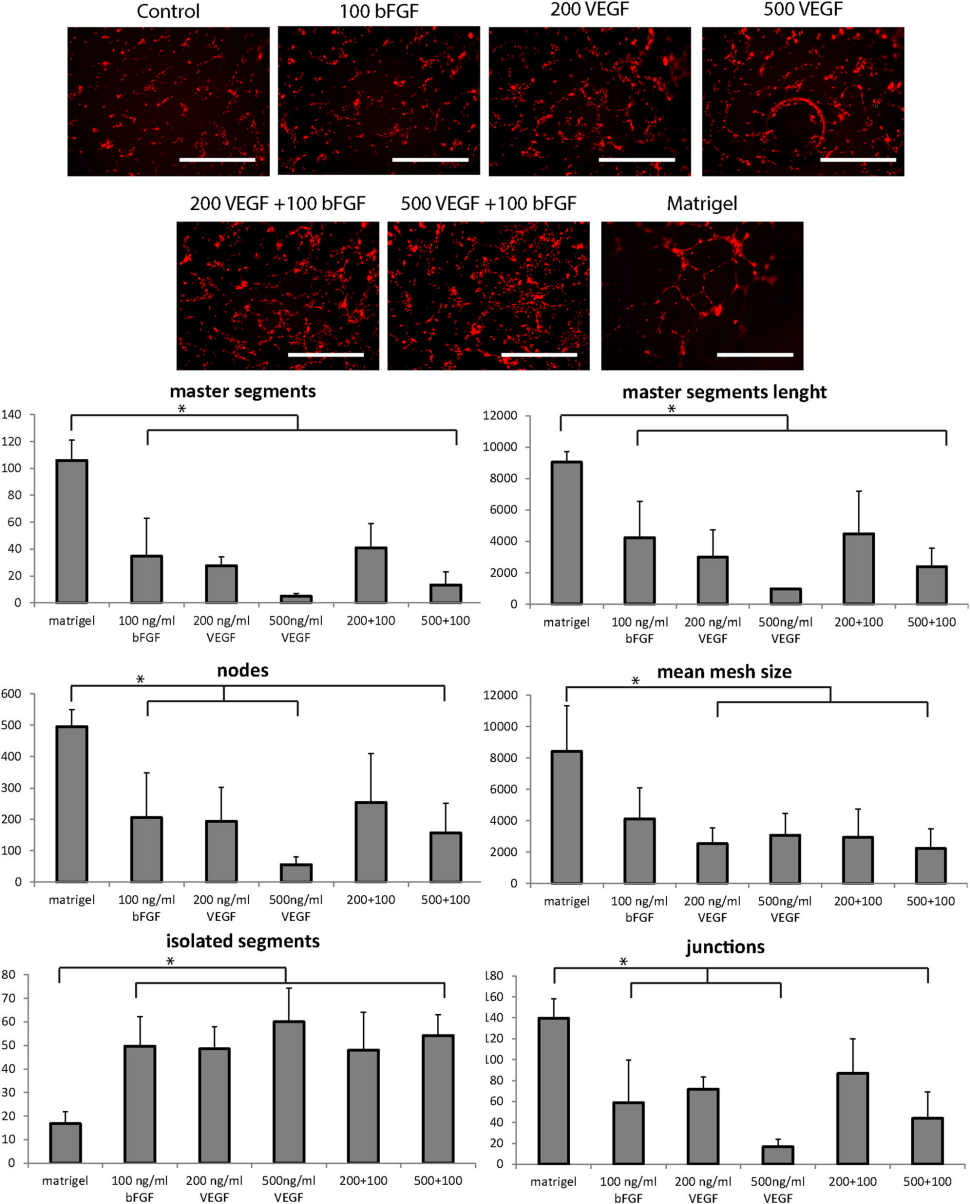
Tube formation assay of HUVEC seeded on vascularization hydrogels at different growth factors loads and combinations thereof. Matrigel is used as positive control and quantification of master segments number, master segments length, nodes, mean mesh size, isolated segments and junctions on tube formation assay of HUVEC seeded on vascularization hydrogels. Significant differences between groups are indicated with an (*) (*p* ≤ 0.05). Scale bar: 1 mm

HUVEC seeded on the surface of the hydrogels adhered to the material in about 4 h, showing a spread morphology and cellular organization within 24 h. The degree of cell organization with respect to preliminary tubular network formation on the surface of the gels was dependent on the growth factor dose embedded within the gel layer (Fig. 3). While in the control without growth factor cobblestone-shaped cells were organized in clusters, when growth factors were present cells were more spread on the gel surface, displayed a more elongated shape and connected to each other with their cytoplasmatic extensions. In growth factor loaded gels, polygonal areas delimited by cells were visible, similar, although much less defined, than the ones formed on Matrigel control gels. Although in none of the conditions we were able to achieve the same degree of tube formation obtained on Matrigel (positive control), it was possible to quantify cellular organization in preliminary tubular structure (Fig. 3).

In all the conditions tested, Matrigel scored the highest in master segments and master segments length, nodes, junctions and mean mesh size, indicating the highest degree of network organization. It also obtained the lowest score in isolated segments, meaning that the great majority of the cells were integrated in the network. Interestingly, hydrogels wthout growth factors were also analysed, but no pattern on these images was detected, thus resulting in no quantification. Overall, a comparable trend emerged from the picture analysis in all the investigated parameters: 100 ng/ml bFGF alone and in combination with the 200 ng/ml VEGF being the conditions that showed an increased tubular network formation in comparison to the 500 ng/ml VEGF alone and in combination with bFGF 100 ng/ml. Remarkably, gels with 100 ng/ml bFGF and 100 ng/ml bFGF + 200 ng/ml VEGF scored no significant difference in comparison to Matrigel regarding nodes, mean mesh size and junction formation. By comparing the combination (bFGF + VEGF) vs. bFGF alone, it seemed that bFGF was the mayor responsible for HUVEC organization on the gel.

**Fig. 4:**
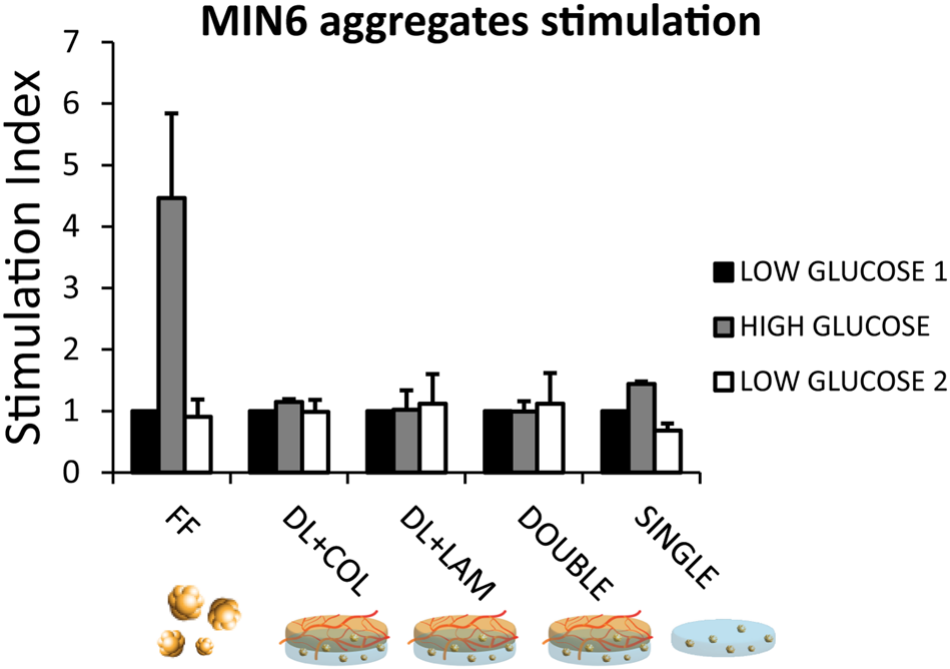
Stimulation index of free-floating MIN6 pseudo-islets, MIN6 aggregates embedded in the single layer PEGDA hydrogel and in a double layered hydrogel without protein addition and in presence of collagen 4 or laminin. Constructs have been incubated in low (1.67 mM) and high (16.7 mM) glucose concentration. Stimulation index is expressed as the ration between the amount of insulin secreted in high glucose condition divided by the basal insulin secretion level (in low glucose condition)

The functionality of MIN6 pseudo-islet embedded in the bilayered constructs was tested and compared to control free-floating aggregates. MIN6 aggregates functionality was heavily reduced compared to the control when aggregates were embedded in the single layer hydrogel (PEGDA only) and completely absent when the embedding layer of PEGDA was crosslinked on top of the vascularization layer. Addition of extracellular matrix proteins as collagen or laminin was not sufficient to rescue pseudo-islets functionality when embedded in the double layer construct (Fig. 4).

**Fig. 5:**
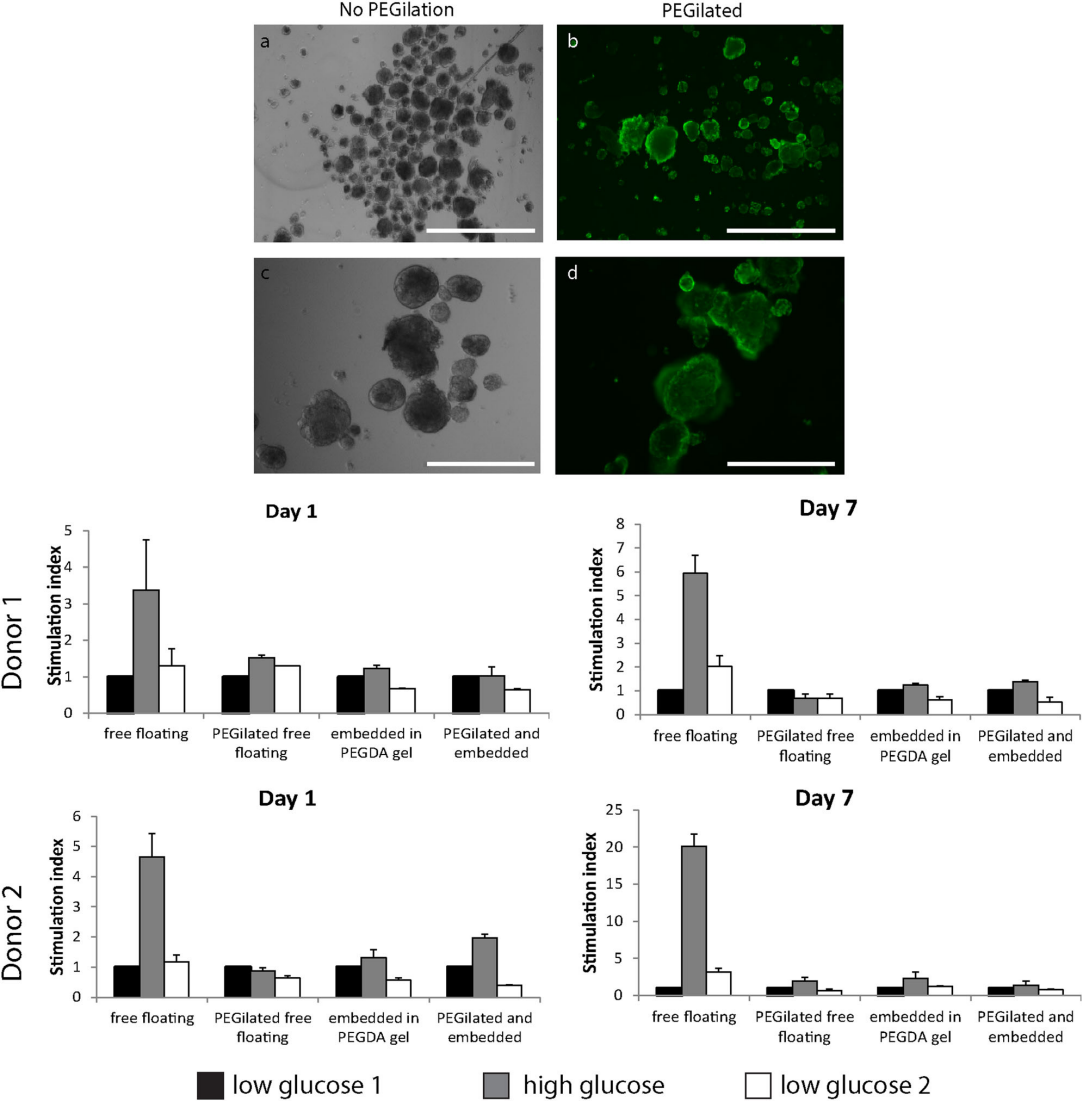
Control (**a**,**c**) and pegilated (**b**,**d**) islet showing a NHS-PEG-biotin layer covalently crosslinked on islet surface proteins and visualized using a streptavidin-FITC conjugate. Scale bar: 1000 µm in the upper row (**a**,**b**) and 400 µm lower row (**c**,**d**). The stimulation index of islet of Langerhans from two donors in basal condition (1.67 mM glucose) and high glucose stimulation (16.7 mM). Free floating islet have been compared to pegilated free floating islet, islet embedded in the construct and islets embedded in the gel after PEGylation. Both at day 1 and day 7 functional response is reduced in all the experimental groups

Successful surface modification with attachment of PEG chains to the amino groups of proteins exposed on the islet of Langerhans extracellular matrix was confirmed by a fluorescent staining appearing on the surface of the islets (Fig. 5).

Functionality of islets of Langerhans from two human donors was analyzed at two time points and compared to the functional response of free floating islets (untreated), PEGylated free floating islets, islets embedded in PEGDA hydrogels and PEGylated islet embedded in the gel. For all donors and at both time points, functional insulin secretion in response to high glucose stimulation was reduced compared to the free floating control. Donor 2 performed overall better with a higher stimulation index. At day 1, PEGylated islets embedded in the gel showed still a functional response. At day 7 all the conditions showed a borderline functional behavior, but the embedding and PEGylation procedure seemed to exert a detrimental effect on islet functionality.

## Discussion

PEGDA is a commonly used matrix for islet of Langerhans encapsulation [34–38]. PEGDA has been widely used in tissue engineering for cell encapsulation in cartilage regeneration [39–41], heart valves regeneration [42], islets [36] or beta cells transplantation [34, 35, 38], and for controlled delivery systems of pro-angiogenic growth factors [17, 18, 43] with promising results. The main advantages of this synthetic matrix are that it is highly defined, with tunable molecular weight, hydrogel mesh size, mechanical properties, water content comparable to soft tissues in the body [44], and ease of functionalization thanks to tunable free binding groups within the matrix [44].

Although widely used, this very versatile system for cell encapsulation also poses limitations. Being PEGDA matrices inert and non-cell adhesive, considerable efforts in functionalizing PEGDA hydrogels with peptides and molecules of the extracellular matrix have been spent in order to confer cell adhesion properties to the material [45, 46]. Although chemical functionalization is pretty straightforward, finding the right cocktail and concentrations of peptides for a specific cell type is not a trivial subject. Weber and coworker [37] functionalized PEG gels with collagen IV, laminin, a combination thereof, or peptides derived from these proteins and found an increase in insulin secretion. Many other adhesion peptides like RGD sequences have been incorporated in PEG gels to improve their adhesion properties [45, 46]. Also matrix metalloprotease sequences have been integrated in the mesh to allow cells to rearrange the synthetic matrix [47]. Another way to increase cell adhesion on PEGDA based hydrogels is to include extracellular matrix molecules like heparin, hyaluronic acid and collagen (or gelatin). Hyaluronic acid and collagen molecules can enhance cell binding via specific receptors like integrins [48] and CD44 [49]. Heparin is a common constituent of the extracellular matrix and is widely used for binding and stabilizing growth factors.

In this study, a bi-layered scaffold concept offers the advantage of combining all the aforementioned tunable properties of PEGDA hydrogels in a unique, multifunctional construct. Non-cell adhesive PEGDA layer mixed with collagen and laminin can be used for islet of Langerhans encapsulation, in the attempt of reducing cell attachment and maintaining islets round morphology. PEGDA layer with the addition of thiol-modified extracellular matrix molecules like heparin, hyaluronic acid and gelatin can serve to increase cell adhesion cues on the hydrogel surface, act as a controlled release system for pro-angiogenic growth factors and ultimately increase blood vessels ingrowth in the construct.

In an alternative approach, the PEGDA layer has been reduced to a conformal coating around the islet surface and embedded into the vascularization layer. In both approaches, both layers were covalently crosslinked to each other in a single multilayered construct using free acryl groups present either at the surface of both gels or on the surface of the islets.

Thiol functionalized heparin, gelatin and hyaluronic acid react spontaneously in mild, physiological conditions with acryl groups on PEGDA forming a stable gel network via a Michael-type addition reaction. This ECM enriched PEGDA gel was able to sustain dual controlled release of VEGF and bFGF for at least 14 days in vitro. The presence of negatively charged glycosaminoglycans assured for slow release of the growth factors from the gel and preservation of their three dimensional structure and activity. The ability of the released growth factor to induce primitive organization of endothelial cells into elongated structures was still lower than Matrigel, but the advantage of this semi-synthetic system is that this is a fully characterized matrix, in which known growth factor cocktails and proteins at known concentration can be embedded.

Further work is required to find an optimal growth factor combination but results reported in literature with similar systems showed promising outcome [17, 18]. In these papers, an increased vessel density with erythrocytes confined in the capillary was found when growth factor loaded hydrogels were implanted in a mouse ear. However, only the induction of vascularization was tested, but no application to an engineered tissue or organ was presented. Furthermore, a more thorough characterization of the thiolated/PEGDA hydrogel mixtures should be considered, including the quantification thiol/acrylates content or the degree of substitution achieved on the different polymers. Although we reduced as much as possible the air exposure of the thiolated GAGs during their reaction with PEGDA, we cannot exclude that disulfide bonds could be formed, which are a notorious challenge to quantify in thiol-ene hydrogel chemistry. Even when this was the case, hydrogels could be formed. This could lead to think that the percentage of such disulfide bonds could be neglected for the purpose of the crosslinking reaction. Furthermore, being these covalently bound hydrogel layers, we could exclude phase separation phenomena that are more characteristic of semi-interpenetrating networks. In vivo studies are necessary to further study growth factor dose and release kinetic on attraction of endothelial cells and revascularization of the embedded islets. Moreover, in vivo degradation studies of this semi-degradable matrix would provide more insights in the release kinetic of the different growth factors and in the timing of the revascularization process.

MIN6 pseudo-islets were used as a model for islets, embedded in the PEGDA layer with or without the addition of collagen IV or laminin. A reduced but still functional response was detected in the single layer gels (thickness of ~1.5 mm), while functionality was lost when a double layer was used, suggesting a limitation in nutrient diffusion through the construct when its thickness is doubled (thickness of ~3 mm) [50, 51]. A higher molecular weight PEGDA could increase the mesh size of the hydrogel and help diminishing nutrient diffusion limitations.

In an attempt to reduce nutrient diffusion limitations in a bi-layered construct, a second strategy was developed and consisted in the conformal coating of islets using a SVA-functionalized PEGDA. SVA would react to free amino groups exposed on proteins on the islet surface. The other acryl functionalized end would react with thiol groups exposed on the vascularization hydrogel. Such a conformal coating on the surface of an islet would only account for a few nanometers in thickness, which should not significantly hamper nutrient diffusion. Moreover, nutrient and oxygen diffusion should be facilitated also by the fact that islets are directly embedded in the vascularization gel. We could speculate that this gel presents higher mechanical properties, in the order of 100–350 kPa [52], compared to UV-crosslinked PEGDA hydrogels (in the order of 50–60 kPa) [53]. A lower concentration of PEGDA crosslinker and the higher molecular weight of the ECM molecules crosslinked within the mesh are expected to account for a larger pore size in the gel mesh and weaker mechanical properties. All these factors are known to result in a hydrogel mesh with bigger distance between crosslinking points, and consequently a larger mesh size, facilitating in this way nutrient diffusion through the matrix, but contributing to weaker mechanical properties. With regard to PEGDA conformal coating, Lee and coworkers also claimed the possibility to achieve immune-protective properties by using this kind of strategy [27–29].

PEGDA binding to amino groups on islet was confirmed by using a NHS-PEG-biotin. NHS-PEG-biotin was used in this case instead of the SVA-PEGDA, because the available biotin functionalization would allow for streptavidin-FITC detection of the successful functionalization. The reactivity of NHS and SVA groups has been reported to be similar [54]. Experimentally, effective PEGylation with SVA-PEGDA was shown by the faster crosslinking speed and increased stiffness of the vascularization gels where PEGylated islets were embedded compared with gels where non-PEGylated islets were embedded. This fact indirectly confirms the effectiveness of SVA-PEGylation.

The functionality of embedded and PEGylated islets in the vascularization layer remained borderline. The functionality was reduced compared to the free floating control condition; only highly functional islets (donor 2, stimulation index > 4 at day1, >20 at day7) were able to still retain a functional profile after PEGylation and embedding. This fact is indicative for a quite harsh PEGylation reaction. Nutrient diffusion limitation is believed to be the main responsible for islet’s reduction in functionality in the bi-layered constructs, but in case of PEGylated islets it might not be the only reason for such reduction in functionality. Although conformal coating creates a very thin layer, SVA-PEGDA can still impair antibodies to interact with islets surface antigens. It might be that a similar limitation occurs for insulin diffusion from the islet. In this case, a concentration curve of different PEGDA/islet ratios during PEGylation or lower SVA-PEGDA molecular weight might help defining more friendly conditions for conformal coating of islets of Langerhans. Another possible explanation might be that crucial proteins for glucose sensing, like GLUT2 receptors might have undergone a conformational change during PEGylation [55] or that the attachment of PEG chains would impede glucose recognition by the channel and its transport into the cytoplasm. Also in this case, milder conditions for PEGDA-SVA conjugation to islets should be investigated to reduce the possibility of surface proteins inactivation. In summary, the concentration of polyethylene glycol coupled on the surface, the reacting groups, the length of the polymeric chain, quantity of islets used in the coupling reaction and the donor variability in islet functionality are all factors that need to be taken into considerations in future studies.

After improving the functionality of the islets embedded in the hydrogel, this system might offer a considerable advantage in view of translation to the clinic, if compared to conventional alginate or Matrigel embedding systems. PEGDA based hydrogels are not degradable, covalently crosslinked hydrogels, which offers a substantial advantage for the reliability of this system in a long term after transplantation, in particular if immunoprotection is desired. Moreover, PEGDA offers the possibility of having complete control on functionalization both of the islet embedding layer as well as the vascularization layer. In contrast to Matrigel, this synthetic tunable matrix can offer full control on the growth factors type and concentration, eliminates the problem of animal origin material and batch-to-batch variation normally encountered when natural polymers are used. The glicosaminoglycans used in the vascularization layer (heparin and hyaluronic acid) are already approved for human use in medical devices, while a point of attention could be the use of gelatin. However, gelatin could be easily substituted by adhesion peptides or human collagen sequences. The stronger mechanical properties of these hybrid hydrogels compared to PEGDA alone would not be detrimental for clinical translation, as shown by the mechanical stability of the constructs developed in this study.

Envisioning a possible clinical translation, this system offers several potential advantages in terms of safety, thanks to the synthetic materials used, control over their production, batch-to-batch variation, and control over their functionalization with human derived proteins or glycosaminoglycans. On the efficacy side, ameliorations in the system are needed to better control islet functionality, hydrogel mesh size, nutrient diffusion and PEGylation conditions and should be implemented before pre-clinical evaluation of the presented hydrogel construct.

## Conclusions

PEGDA based hydrogels represent a versatile matrix for cell embedding. The addition of extracellular matrix proteins and thiol-modified glycosaminoglycans provided cell adhesion properties and growth factor binding and release capability to the otherwise inert PEGDA matrix. These results show the potential of the vascularization layer to attract and induce organization of an endothelial cell layer in close proximity to islets of Langerhans.

More research is required in tuning mesh size properties in the PEGDA layer for islets of Langerhans embedding, since limited nutrient diffusion still seems to remain the biggest limiting factor in islet functionality. The alternative PEGylation approach reduces the thickness of the PEG layer surrounding the islets. However, the PEGylation procedure might irreversibly change the three dimensional conformation of proteins exposed on the surface of the islets.
